# Supplementary material for: Adaptation and Dissemination of Korean Medicine Clinical Practice Guidelines for Traffic Injuries
Source: Healthcare (Basel). 2022 Jun 22;10(7):1166. doi: 10.3390/healthcare10071166 (PMC9316782; doi:10.3390/healthcare10071166)
Supplement: Supplementary file 1 [file healthcare-10-01166-s001.zip › Supplementary Figure S1.pdf]

교통사고 상해증후군 한의표준임상진료지침

Guideline Center for Korean Medicine
GCKoM
한의표준임상진료지침 개발사업단

Clinical algorithm for traffic injuries

Grade Ⅰ
- Complaint of neck pain, stiffness or tenderness only
- No physical sign

Grade Ⅱ
- Complaint of neck pain, stiffness
- Musculoskeletal signs include decreased Range of movement

Grade Ⅲ
- Neck complaint and neurological sign
- Neurological signs include decreased or absent tendon reflex, weakness and sensory deficits

Grade Ⅳ
- Neck Complaint and fracture or dislocation

Korean medicine alone — NO — Integrative treatment — YES — Western medicine

Korean medicine alone
- Grade Ⅰ, Ⅱ
- Grade Ⅲ (mild)
- Grade Ⅲ (severe) AND sequelae of Grade Ⅳ
- Post-traumatic stress disorder

Western medicine
- Grade Ⅲ (severe) and Grade Ⅳ
- Surgical treatment : Imaging examination and consult for emergency surgery
- Non-surgical treatment : Western medicine and injection treatment. Fracture related cast treatment

Moxibustion
- Alone treatment for neck/Low back pain **(C/Very low)**
- Combination treatment for neck pain **(C/Low)**
- Combination treatment for Low back pain **(C/Very low)**
  - Ashi point and combine with cupping
  - Consider precautions such as infection during venesection

Cupping
- Alone or Combination treatment for neck/Low back pain **(C/Very low)**
  - Ashi point and combine with cupping
  - Consider precautions such as infection during venesection

Physiotherapy
- Combination treatment with ICT, TENS, MW, SSP **(C/Very low)**
  - Do-in exercise therapy, Musculotendinous releasing therapy

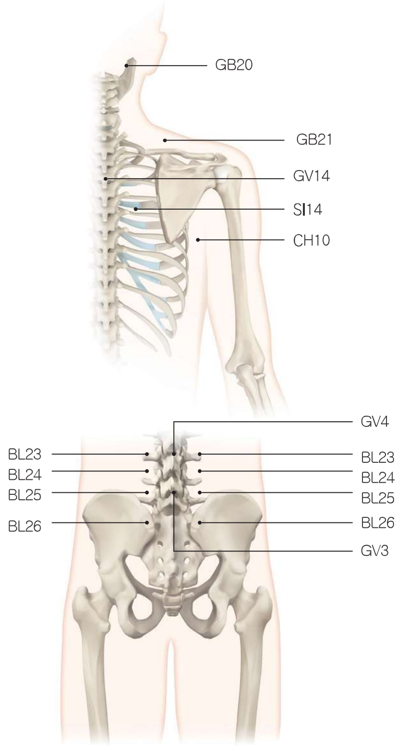

Chuna & Pharmacopuncture
- Chuna and Pharmacopuncture combination treatment for neck/Low back pain **(C/Very low)**
- Pharmacopuncture with conventional treatment with Chuna for neck pain **(C/Low)**

  Chuna and conventional treatment with pharmacopuncture for neck pain **(B/moderate)**

Chuna
- Alone treatment for neck/Low back pain **(C/Very low)**
- Combination treatment for neck pain **(C/Low)**
- Combination treatment for Low back pain **(C/Low)**
  - Consider simple Chuna technique (fascia, joint movement, joint distraction) and complex Chuna technique (joint correction) as chuna for pain and limited range of motion.

Traffic injury → Pattern syndrome

瘀血
- Consider primary

痰飮 **氣滯** **血虛** **氣虛**

- Detailed diagnoses are made based on each patient's situation or the opinion of a korean medicine doctor.

Electroacupuncture/ Acupuncture
- Combination treatment with acupuncture for neck pain **(B/Moderate)**

  Combination treatment with electroacupuncture for neck pain **(B/Moderate)**

  Combination treatment with MSAT for neck pain **(B/Moderate)**
- Combination treatment with acupuncture for Low back pain **(C/Very low)**

  Combination treatment with eletroacupuncture for Low back pain **(C/Low)**
  - SI14, GB20, GB21, TE05, GV14, and Ashi-point
  - Hua-Tuo-Jia-Ji-Xue, BL25, BL23 and Ashi-point
  - Concurrent eletroacupuncture for high-intensity pain

Pharmacopuncture
- Alone treatment for neck/Low back pain **(C/Very low)**
- Combination treatment for neck pain **(B/Moderate)**
- Combination treatment for Low back pain **(C/Very low)**
  - Consider using bee venom, hwalyeolhwaeo, anti-inflammatory for analgesia, and muscle relaxation.
  - Neck pain : GV16, GB20, GB21, GV14
  - Low back pain : BL23, BL24, BL25, GV3, GB30, Hua-Tuo-Jia-Ji-Xue

  ※ Bee veenom is must performed after skin test

Herbal medicine
- Alone or combination treatment with herbal medicine for neck/Low back pain **(C/Very low)**
  - Detailed prescription is possible according to the individual patient's pattern syndrome

Notice The clinical practice guidelines are developed systematically to help Korean medcine doctors and patients decide on appropriate medical services, and can be used as a reference in the clinical field. This guideline was certified through the Korean standard clinical practice guideline development project.

Guideline Center for Korean Medicine
GCKoM
한의표준임상진료지침 개발사업단
