# Supplementary material for: Adaptation and Dissemination of Korean Medicine Clinical Practice Guidelines for Traffic Injuries
Source: Healthcare (Basel). 2022 Jun 22;10(7):1166. doi: 10.3390/healthcare10071166 (PMC9316782; doi:10.3390/healthcare10071166)
Supplement: Supplementary file 1 [file healthcare-10-01166-s001.zip › Supplementary Figure S2.pdf]

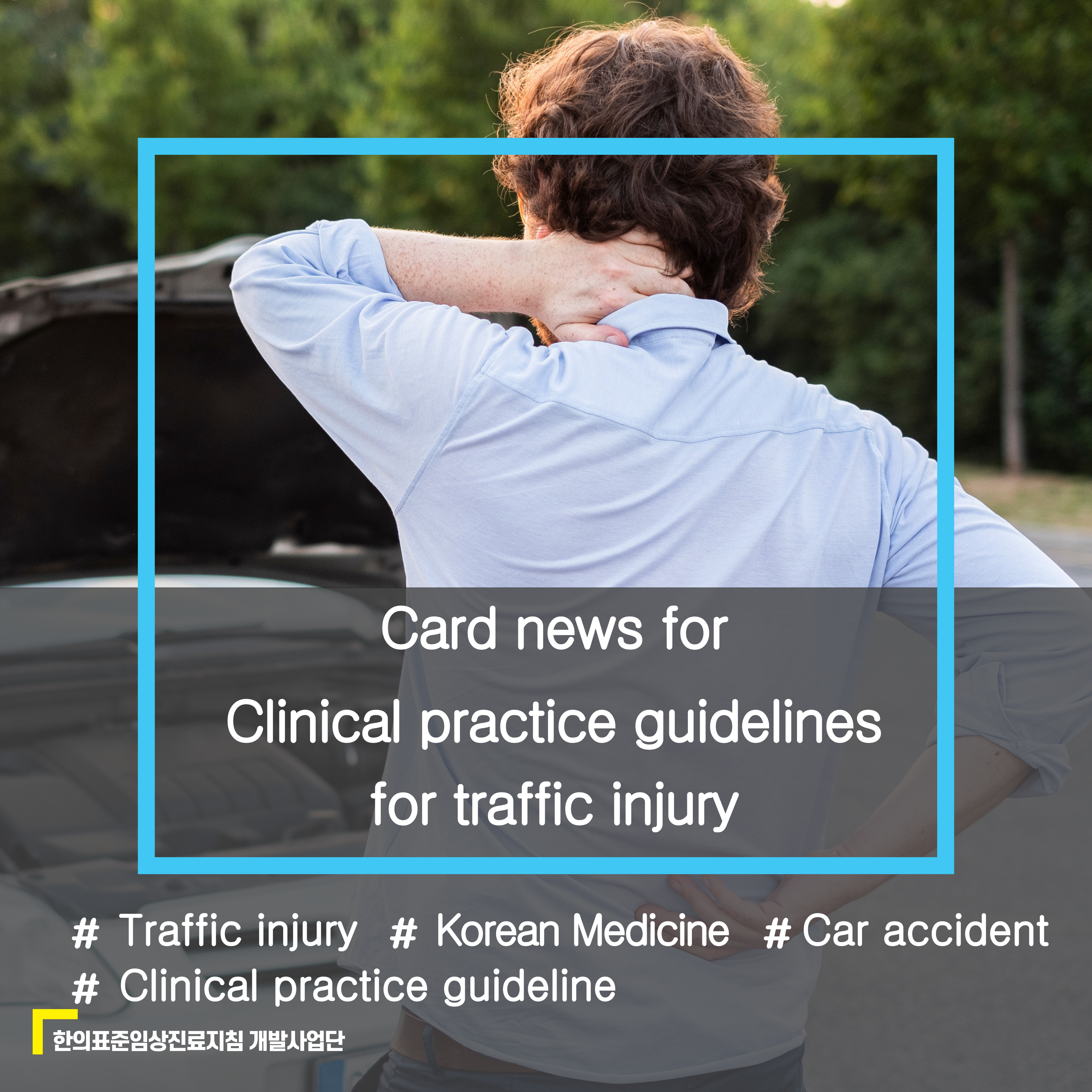
Card news for
Clinical practice guidelines
for traffic injury
Traffic injury # Korean Medicine # Car accident
Clinical practice guideline
한의표준임상진료지침 개발사업단

Traffic injury

As the impact of an accident is transmitted to the body, various ligaments and muscles around the spine are damaged, causing various musculoskeletal symptoms such as neck pain, stiffness, back pain, numbness, headache, dizziness, fatigue, and depression.

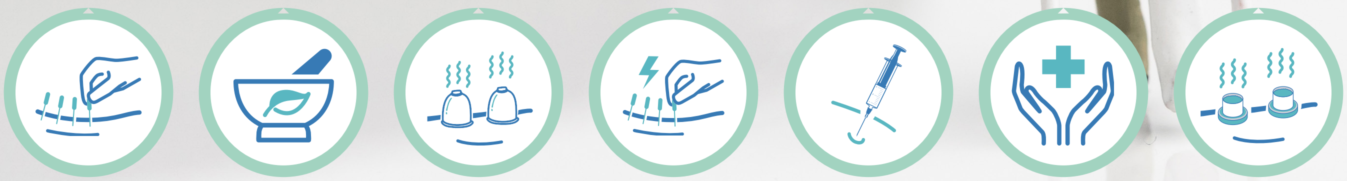

The goal of Korean medicine treatment is to recover the damaged area by removing the blood stagnation (瘀血).

What is the **satisfaction level** of patients who received **Korean medicine treatment** after a traffic injury?

228 (27%) of 845 patients were extremely satisfied with Korean medicine treatment, 468 (55.4%) were satisfied, 138 (16.3%) were normal, 4 (0.5%) were dissatisfied, and 6 (0.7%) were extremely dissatisfied

한의표준임상진료지침 개발사업단

*Source: Clinical Practice Guideline for Traffic Injury in Korean Medicine

한의표준임상진료지침 개발사업단

Electroacupuncture

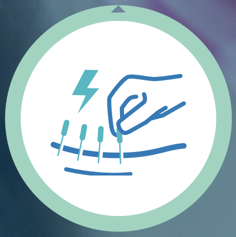

evidence level ★★★★☆ Grade

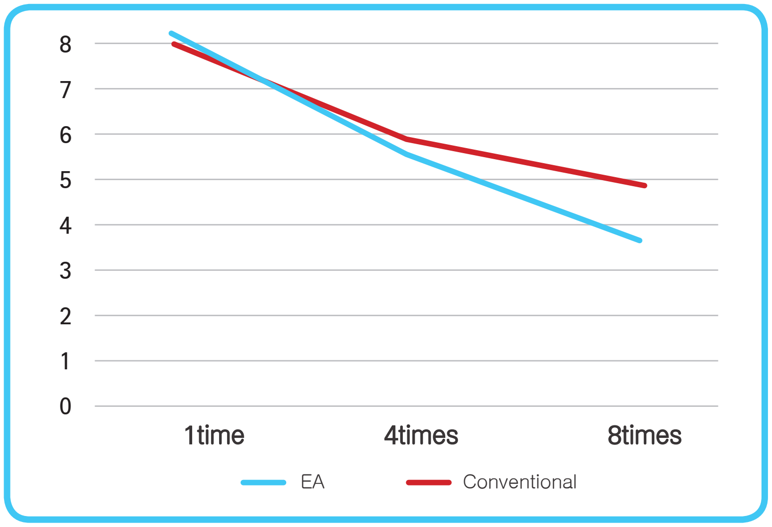

Neck pain intensity change in 4 weeks

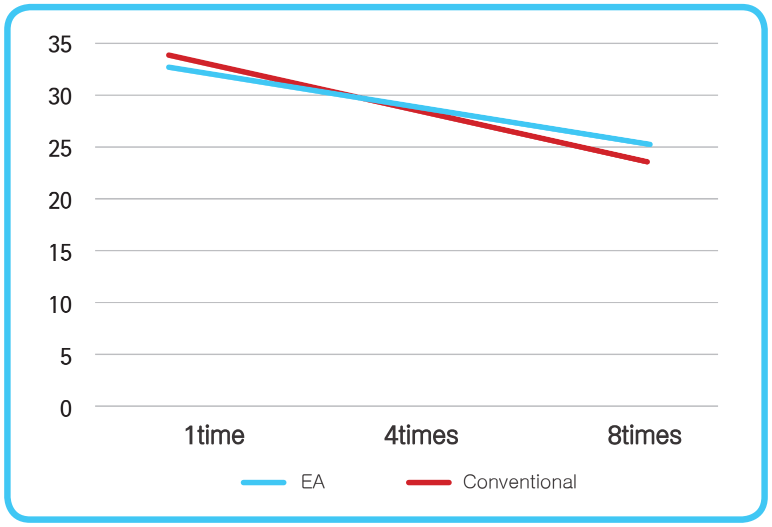

Neck disability index improvement in 4 weeks

– Electroacupnucture was **effective** for neck pain and functional improvement after traffic injury

– **Analgesic effect** through electroacupuncture stimulation

한의표준임상진료지침 개발사업단

*Source: Clinical Practice Guideline for Traffic Injury in Korean Medicine

Motion-style acunpuncture technique

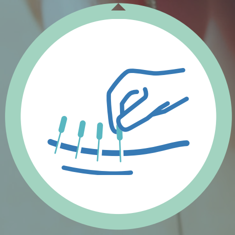

evidence level ★★★★☆ Grade 👍👍👍👍☆

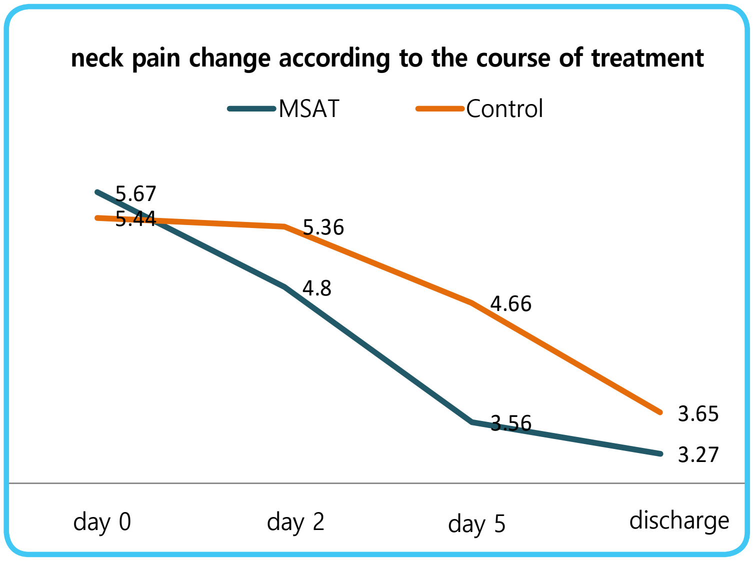

MSAT and concurrent Korean medicine treatment were combined, pain alleviation and recovery of the neck's range of motion were decrese faster than when concurrent Korean medicine treatment performed alone.

MSAT

Motion-Style Acupuncture Technique (MSAT) is one of the acupuncture techniques used to reduce musculoskeletal pain in Korean oriental medicine, which combines simultaneous breathing and exercise activities in acupuncture treatment.

*Source: Clinical Practice Guideline for Traffic Injury in Korean Medicine

한의표준임상진료지침 개발사업단

Pharmacopuncture

evidence level ★★★★☆

Grade 👍👍👍👍

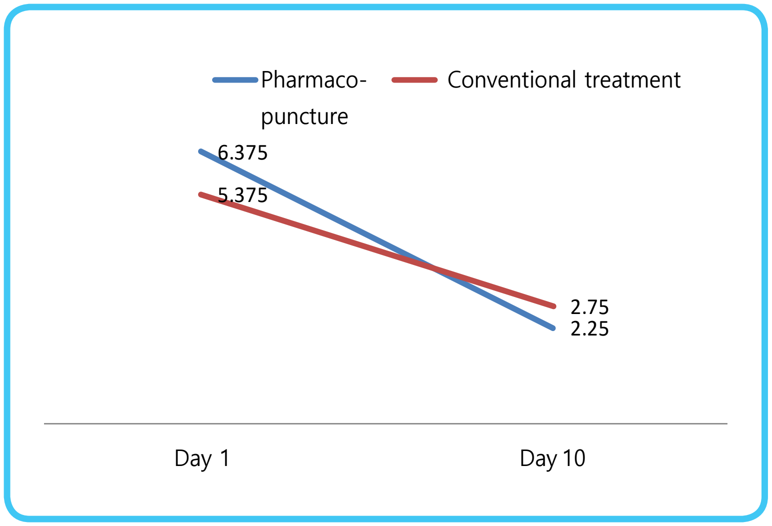

Neck pain intensity change

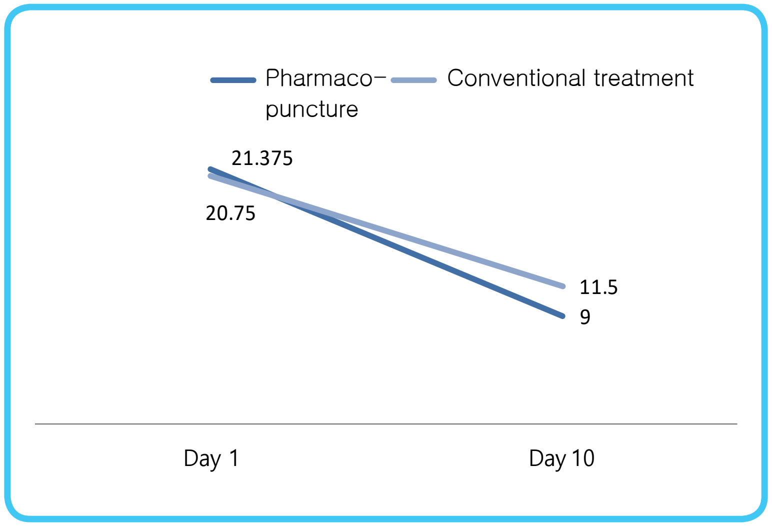

Neck disability index improvement

Pharmacopuncture is effective for neck pain and function improvement with traffic injury!

한의표준임상진료지침 개발사업단

*Source: Clinical Practice Guideline for Traffic Injury in Korean Medicine

한의표준임상진료지침 개발사업단

Chuna maunal therapy

evidence level ★★★☆☆

Grade 👍👍👍

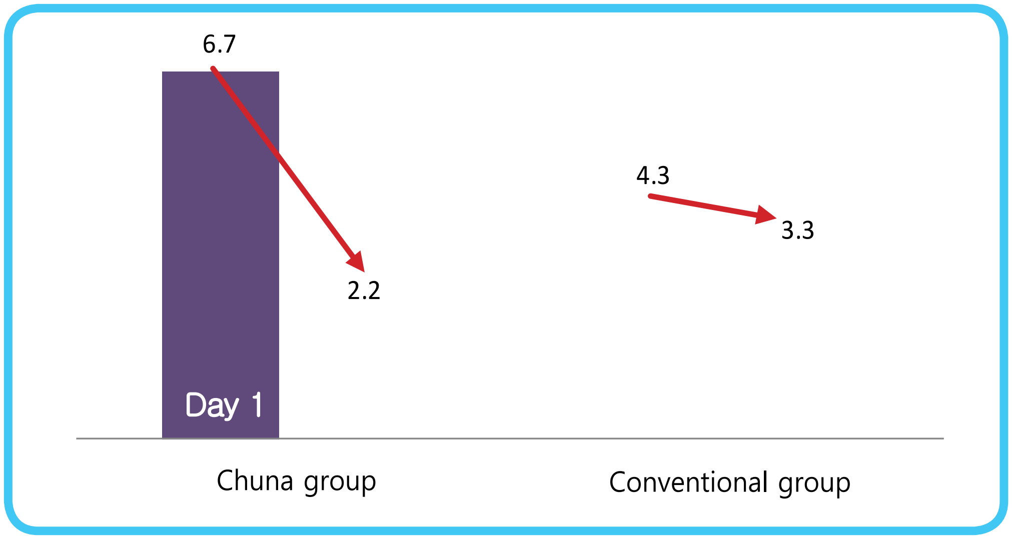

Changes pain intensity

Chuna manual therapy is effective in decreasing pain intensity and improving function of low back after traffic injury.

*Source: Clinical Practice Guideline for Traffic Injury in Korean Medicine

한의표준임상진료지침 개발사업단

Pharmacopuncture + chuna manual therapy

Evidence level

Grade

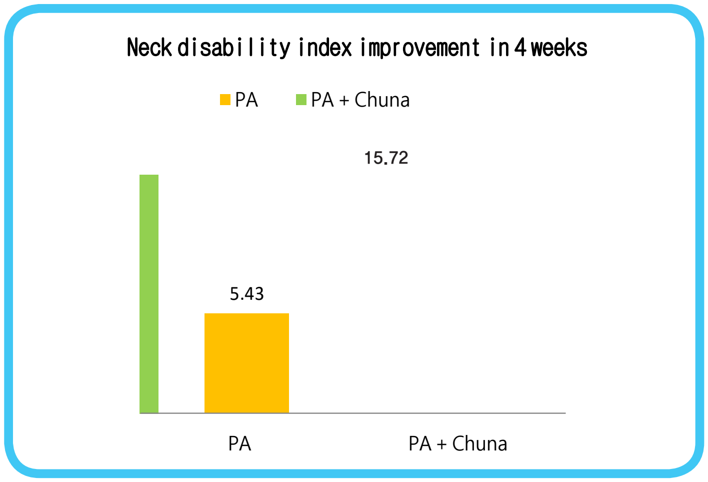

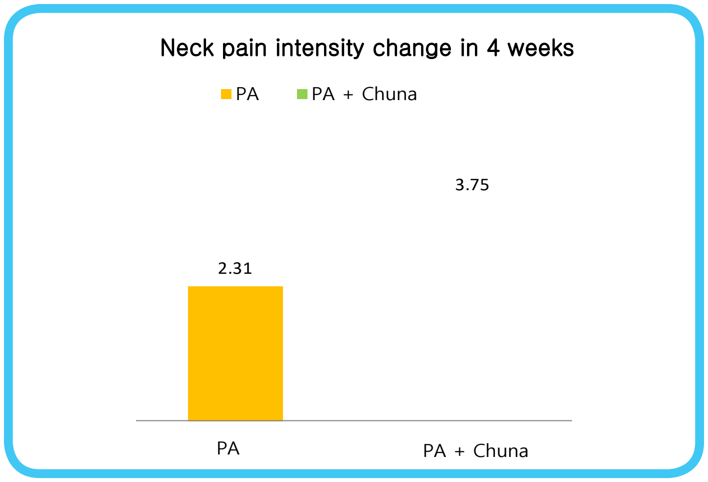

When acupuncture and chuna treatment were combined, it was more effective in reducing neck pain and improving functions than treating acupuncture only

*Source: Clinical Practice Guideline for Traffic Injury in Korean Medicine

After traffic injury,

Acupuncture, electroacupuncture, pharmacopuncture, chuna, etc. are effective!

Acupuncture

Electro-Acupuncture

Pharma-copuncture

Chuna Manual Therapy

Moxibustion

Pharmacopuncture + Chuna manual Therapy

Cupping

Herbal medicine

Cupping and herbal medicine treatment are also effective!

If you feel pain and have limited movement after a car accident, visiting **the nearest korean medicine clinic/hospital** for counseling is the shortcut to a quick return to daily life!

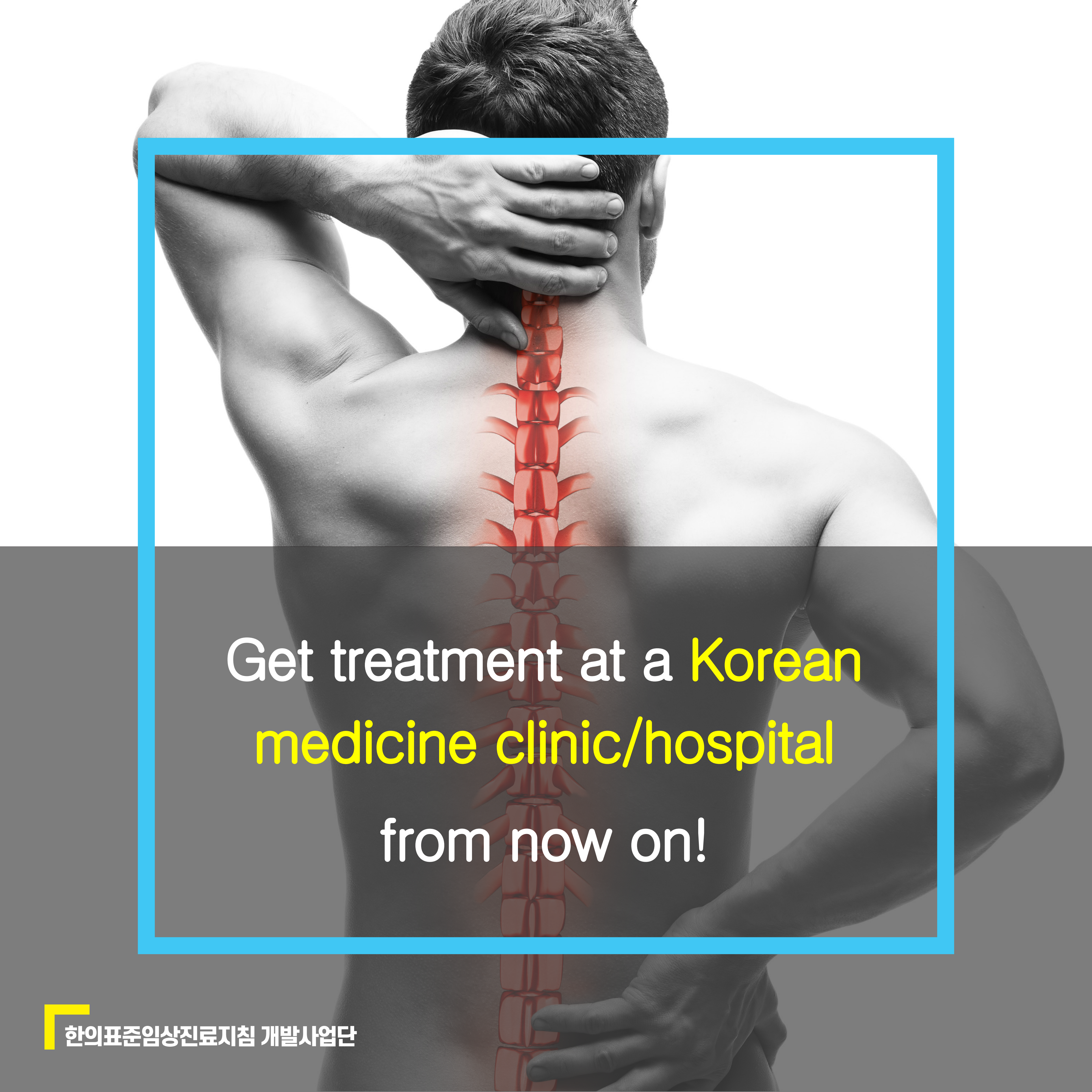
Get treatment at a Korean medicine clinic/hospital
from now on!
한의표준임상진료지침 개발사업단
