# Supplementary material for: Adaptation and Dissemination of Korean Medicine Clinical Practice Guidelines for Traffic Injuries
Source: Healthcare (Basel). 2022 Jun 22;10(7):1166. doi: 10.3390/healthcare10071166 (PMC9316782; doi:10.3390/healthcare10071166)
Supplement: Supplementary file 1 [file healthcare-10-01166-s001.zip › Supplementary Figure S3.pdf]

What is Traffic injury ?

Traffic Accident Injury Syndrome refers to systemic symptoms such as skeletal damage, soft tissue damage, internal bruises, and mental pain that occur after a traffic accident.

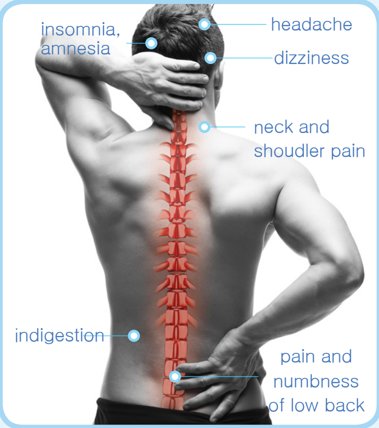

As the impact of an accident is transmitted to the body, various ligaments and muscles around the spine are damaged, causing various musculoskeletal symptoms such as neck pain, stiffness, back pain, numbness, headache, dizziness, fatigue, and depression.

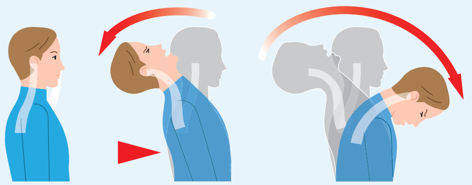

The goal of Korean medicine treatment is to recover the damaged area by removing the **blood stagnation (瘀血).**

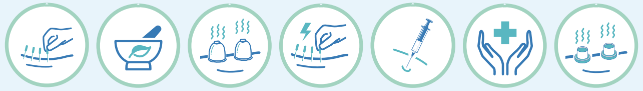

Acupuncture | Herbal medicine | Cupping | Electro–acupuncture | Pharmacopuncture | Chuna Manual Therapy | Moxibustion

The clinical practice guidelines are developed systematically to help Korean medcine doctors and patients decide on appropriate medical services, and can be used as a reference in the clinical field. This guideline was certified through the Korean standard clinical practice guideline development project.

▶ Prevention of traffic injury

You can prevent a whiplash injury from a traffic injury by:
The top of the driver's head should be in line with the top of the head rest, preferably no more than 2 to 5 cm between the back of the head and the headrest.

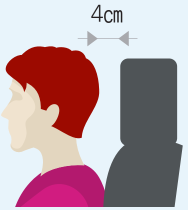

▶ Daily management of traffic injury

- After a car accident, injured muscles can become stiff and weak if not used. This can increase pain and delay recovery.
- Sitting for long periods of time in one position is not recommended.
- Stand up and stretch regularly.
- Adjust your seat when driving so that your elbows and knees are bent.
- Avoid strenuous exercise for the first few weeks to prevent further injury.

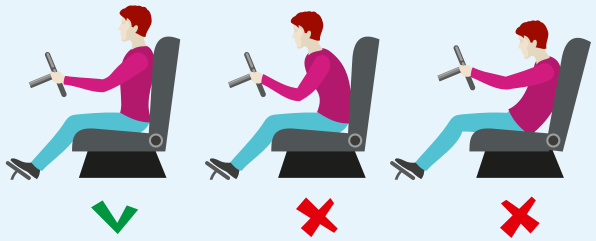

Our hospital complies with the standard clinical practice guidelines of Korean Medicine for Traffic Injury from the diagnosis, treatment selection, and treatment evaluation of traffic accident injuries.

MEMO

교통사고 상해증후군

한의표준임상진료지침

Korean Medicine Clinical Practice Guideline

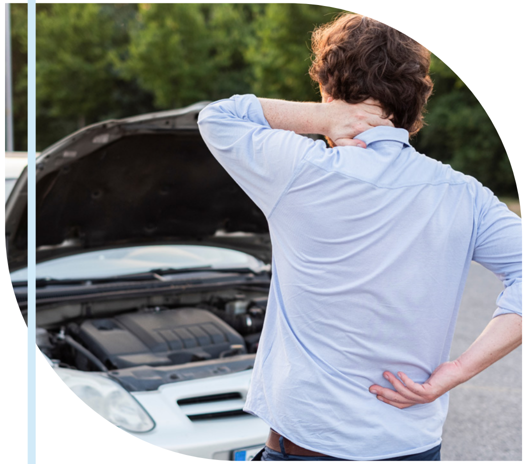

Korean Medicine Clinical Practice Guideline

Guideline Center for Korean Medicine
GTKoM
한의표준임상진료지침 개발사업단

Acupuncture and electroacupuncture are effective for after-effects of traffic injuries.

Acupuncture

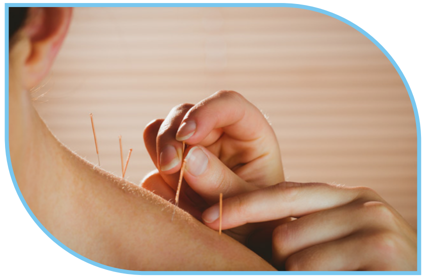

When acupuncture is treated together with conventional treatment, it is effective in improving neck pain and joint range of motion.

Electroacupuncture

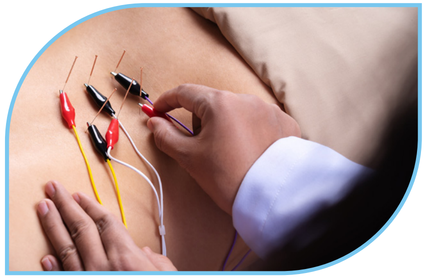

In order to obtain an analgesic effect through low-frequency electroacupuncture stimulation, it is recommended to use strong stimulation to the extent that the patient can tolerate and contract the muscles and muscle tension or chronic pain suddenly worsens, high-frequency-low-intensity electroacupuncture can provide muscle relaxation and analgesia.

Acupoint

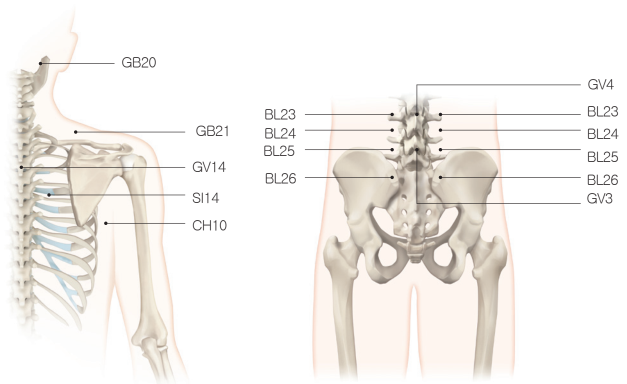

Pharmacopuncture

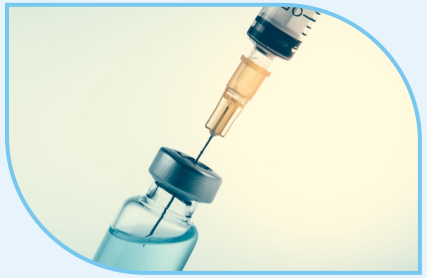

For Pharmacopuncture, consider using bee venom, hwalyeolhwaeo (活血化瘀), anti-inflammatory (消炎) for analgesia, and muscle relaxation.

It is recommended to use herbal acupuncture along with Korean medicine concurrent therapy.

Moxibustion

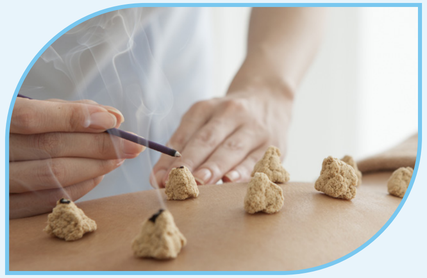

It can also be considered to combine moxibustion with conventional Korean Medicine treatment.

Chuna Manual Therapy

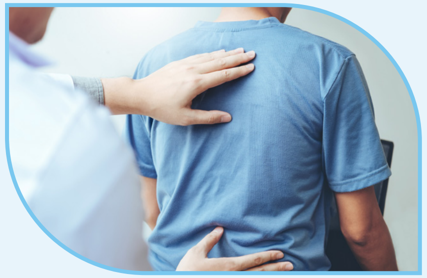

Chuna Manual Therapy is effective in relieving the pain intensity and improving function of neck and back pain.

Herbal Medicine

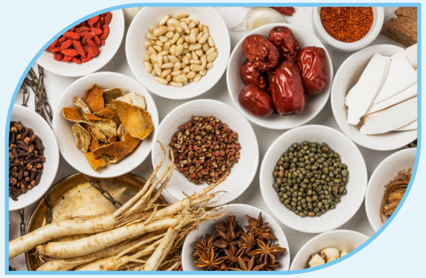

For the treatment of traffic injury, the basic prescriptions are Dangguisu-san (當歸鬚散), Ojeok-san (五積散), Galgeun-tang (葛根湯), and Hoesoo-san (回首散). Depending on the condition and characteristics, you can receive an appropriate prescription.

Physiotherapy

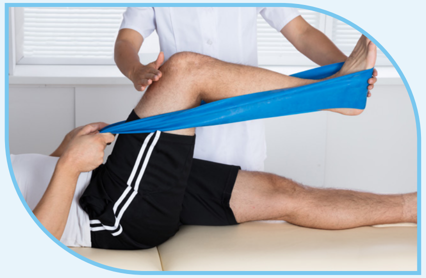

Considering the patient's characteristics and condition, electrical stimulation therapy, phototherapy, hydrotherapy, and Do-in exercise therapy may be considered according to the judgment of the Korean medicine doctor.

Cupping

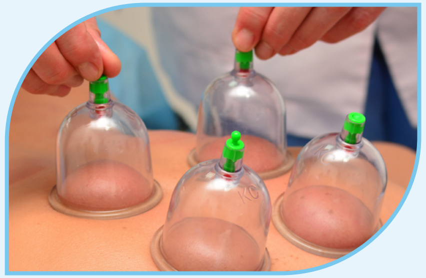

It is recommended to perform acupuncture and cupping together after a traffic injury.
