# Supplementary material for: Adaptation and Dissemination of Korean Medicine Clinical Practice Guidelines for Traffic Injuries
Source: Healthcare (Basel). 2022 Jun 22;10(7):1166. doi: 10.3390/healthcare10071166 (PMC9316782; doi:10.3390/healthcare10071166)
Supplement: Supplementary file 1 [file healthcare-10-01166-s001.zip › Supplementary Table S1.pdf]

Supplementary Table S1. The member list of the committees

Committees	Position	Name	Institution	Region	Role/Specialty
Development Committees	member	Byung-Cheul Shin	Pusan National University Korean Medicine Hospital	Yangsan	Principal investigator (specialist of Korean Medicine Rehabilitation)
		Eui-Hyoung Hwang	Pusan National University Korean Medicine Hospital	Yangsan	hands-on worker of development CPG /Methodology expert (specialist of Korean Medicine Rehabilitation)
		Eun-Jung Lee	Daejeon University Korean Medicine Hospital	Daejeon	Development of CPG (specialist of Korean Medicine Rehabilitation)
		Jae-Heung Cho	Kyung Hee University Korean Medicine Hospital	Seoul	Development of CPG (specialist of Korean Medicine Rehabilitation)
		In-Hyuk Ha	Jaseng Korean Medicine Hospital	Seoul	Development of CPG (specialist of Korean Medicine Rehabilitation)
		Sung-Chul Kim	Wonkwang University Gwangju Korean Medicine Hospital	Gwangju	Development of CPG (specialist of Korean Medicine Rehabilitation)
		Jae-Hong Kim	Dongshin University Korean Medicine Hospital	Naju	Development of CPG (specialist of Korean Medicine Rehabilitation)
		Jong-In Kim	Kyung Hee University Korean Medicine Hospital	Seoul	Development of CPG (specialist of Acupuncture & Moxibustion Medicine)
		Hyung-Won Kang	Wonkwang University Korean Medicine Hospital	Iksan	Development of CPG (specialist of Korean Medicine Neuropsychiatry)
		Ki-Byung Kim	Chamsol Korean Medicine Clinic	Daejeon	Development of CPG (Korean Medicine practitioner)
Review and advisory committee	member	Youn-Seok Ko	College of Korean Medicine, Woosuk University (Korean Society of Chuna Manual Medicine for Spine & Nerves)	Jeonju	Review (clinical specialist - specialist of Korean Medicine Rehabilitation)
		Seung-Ryong Yeom	College of Korean Medicine, Wonkwang University (The Society of Korean Medicine Rehabilitation.)	Iksan	Review (clinical specialist - specialist of Korean Medicine Rehabilitation)
		Jong-Uk Kim	College of Korean Medicine, Woosuk University (Korean Acupuncture & Moxibustion Medicine Society)	Jeonju	Review (clinical specialist - specialist of Acupuncture & Moxibustion Medicine)
		Sung-Youl Choi	College of Korean Medicine, Gacheon University (Korean Society of Oriental Neuropsychiatry)	Incheon	Review (clinical specialist - specialist of Korean Medicine Neuropsychiatry)
		Jae-Won Yang	Korean pharmacopuncture institute	Seoul	Delphi research and Review (advice and review on medical reality)
		Ji-Hun Park	Society of sports Korean Medicine	Ansan	Delphi research and Review (advice and review on medical reality)
		Jun-Hwan Lee	Korea Institute of Oriental Medicine	Daejeon	Review of Level of Evidence and Grade of Recommendation (Methodology expert)
		Chang-Sop Yang	Korea Institute of Oriental Medicine	Daejeon	Review of Level of Evidence and Grade of Recommendation (Methodology expert)
		Yun-Yeop Cha	Sanji University Korean Medicine Hospital	Wonju	Review (clinical specialist - specialist of Korean Medicine Rehabilitation)
	Practitioner panel	Min-Kyu Kim	Jaseng Korean Medicine Hospital	Seoul	Agreement (RAM) and review
	Practitioner panel	Ha-Neul Kim	Jaseng Korean Medicine Hospital	Seoul	Agreement (RAM) and review

CPG; Clinical Practice Guideline, RAM; RAND-UCLA Appropriateness Method
